# Targeting hypoxic cancer stem cells (CSCs) with Doxycycline: Implications for optimizing anti-angiogenic therapy

**DOI:** 10.18632/oncotarget.18445

**Published:** 2017-06-12

**Authors:** Ernestina Marianna De Francesco, Marcello Maggiolini, Herbert B. Tanowitz, Federica Sotgia, Michael P. Lisanti

**Affiliations:** ^1^ Department of Pharmacy, Health and Nutritional Sciences, University of Calabria, Rende, Italy; ^2^ The Paterson Institute, University of Manchester, Withington, United Kingdom; ^3^ Departments of Pathology and Medicine, Albert Einstein College of Medicine, Bronx, NY, USA; ^4^ Translational Medicine, School of Environment and Life Sciences, Biomedical Research Centre (BRC), University of Salford, Greater Manchester, United Kingdom

**Keywords:** anti-oxidant, anti-angiogenic therapy, cancer stem-like cells (CSCs), chronic hypoxia, Doxycycline

## Abstract

Here, we report new mechanistic insight into how chronic hypoxia increases ‘stemness’ in cancer cells. Using chemical inhibitors, we provide direct experimental evidence that ROS production and mitochondrial biogenesis are both required for the hypoxia-induced propagation of CSCs. More specifically, we show that hypoxic CSCs can be effectively targeted with i) simple mitochondrial anti-oxidants (Mito-TEMPO) and/or ii) inhibitors of mitochondrial biogenesis (Doxycycline). In this context, we discuss the idea that mitochondrial biogenesis itself may be a primary driver of “stemness” in hypoxic cancer cells, with metabolic links to fatty acid oxidation (FAO). As Doxycycline is an FDA-approved drug, we propose that it could be re-purposed to target hypoxic CSCs, either alone or in combination with chemotherapy, i.e., Paclitaxel. For example, we demonstrate that Doxycycline effectively targets the sub-population of hypoxia-induced CSCs that are Paclitaxel-resistant, overcoming hypoxia-induced drug-resistance. Finally, anti-angiogenic therapy often induces tumor hypoxia, allowing CSCs to survive and propagate, ultimately driving tumor progression. Therefore, we suggest that Doxycycline could be used in combination with anti-angiogenic agents, to actively prevent or minimize hypoxia-induced treatment failure. In direct support of this assertion, Paclitaxel is already known to behave as an angiogenesis inhibitor.

## INTRODUCTION

Hypoxia in the tumor microenvironment is a critical negative prognostic factor that ultimately promotes cancer progression, tumor recurrence and distant metastasis, as well as chemo- and radio-resistance [1, 2]. Therefore, many medicinal chemists, pharmacologists, biologists and clinicians have all sought to invent new “hypoxia-specific” therapeutics to target the hypoxic tumor microenvironment [3, 4]. However, these “hypoxia-specific” strategies and anti-cancer drugs still remain elusive.

Perhaps another approach would be to better understand what are the basic cellular factors that make hypoxia such a strong driver of a lethal tumor microenvironment. For example, it is known that hypoxia can also induce stem cell characteristics in cancer cells [1–4]. Thus, an increase in “stemness” may actually explain the clinical association of hypoxia with a poor prognosis and drug-resistance [1–7].

Here, we provide further mechanistic evidence to support the role of hypoxia in driving CSC propagation. In addition, we show that chronic hypoxia in CSCs induces a specific stress response, which leads to i) increased ROS production and ii) elevated mitochondrial biogenesis. Remarkably, treatment of these hypoxic CSCs with ROS scavengers (Mito-TEMPO) [8] or an FDA-approved inhibitor of mitochondrial biogenesis (Doxycycline) [9–11] effectively blocks the propagation of hypoxic CSCs.

We discuss the possibility that Doxycycline could be re-purposed as an anti-cancer agent to specifically target hypoxic CSCs, either alone or in combination with other chemo-therapeutic agents, such as Paclitaxel. Moreover, we directly demonstrate that Doxycycline effectively targets the sub-fraction of hypoxic CSCs that are Paclitaxel-resistant.

## RESULTS

### Chronic hypoxia stimulates mitochondrial biogenesis and CSC propagation

**Figure 1:**
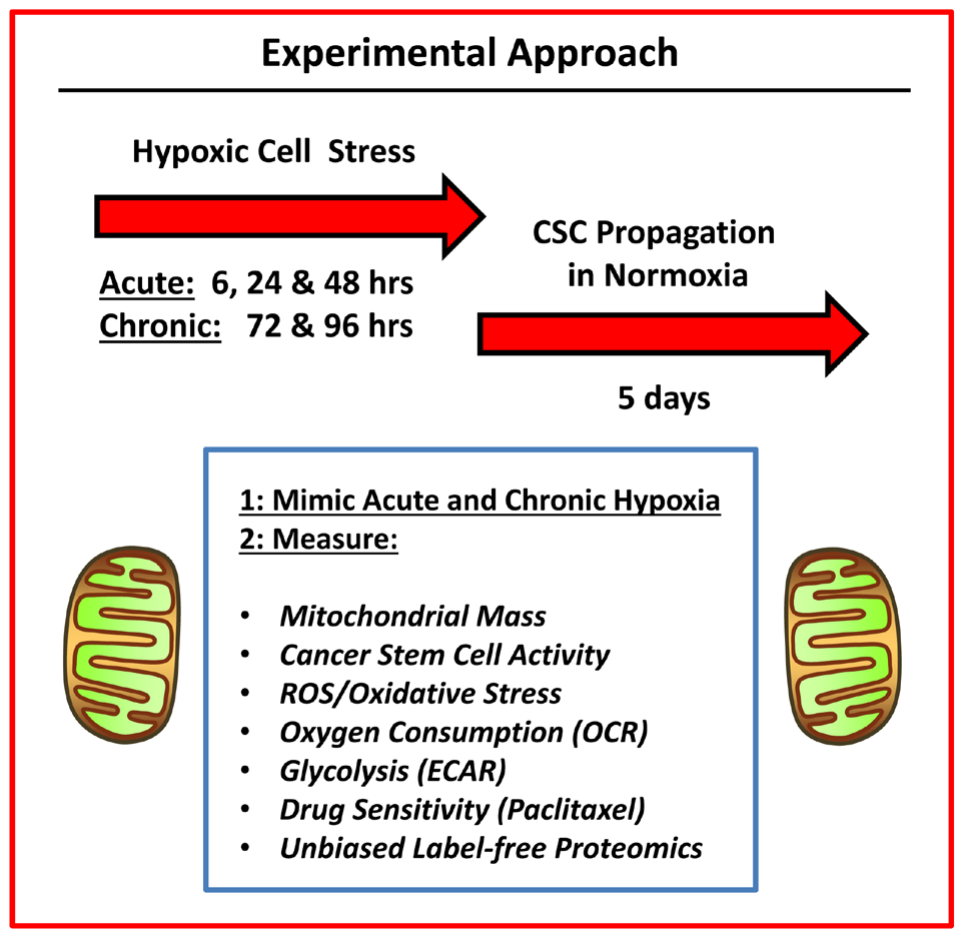
Experimental approach To understand the mechanism(s) underpinning the effects of hypoxic stress on CSC propagation, we used an unbiased metabolic phenotyping approach. Briefly, MCF7 cells were first subjected to acute hypoxia (for 6, 24 and 48 hrs) or chronic hypoxia (for 72 and 96 hrs) grown as monolayers. Then, the hypoxic cells were harvested and subjected to anchorage-independent growth assays (mammosphere formation), to measure cancer stem cell activity. A variety of other metabolic parameters were also quantitated to pinpoint possible targets for therapeutic interventions.

In this report, we systematically examine the role of chronic hypoxia and oxidative stress in the propagation of breast CSCs, using a human tumor cell-line (MCF7 cells) — as a model system (summarized in Figure 1).

As a first step, MCF7 cell monolayers were subjected to hypoxia (1% oxygen) for increasing periods of time (0, 6, 24, 48, 72 and 96 hours). MCF7 cell monolayers were then trypsinized and subjected to FACS analysis with MitoTracker Deep-Red-FM as a probe, to estimate their mitochondrial mass.

**Figure 2:**
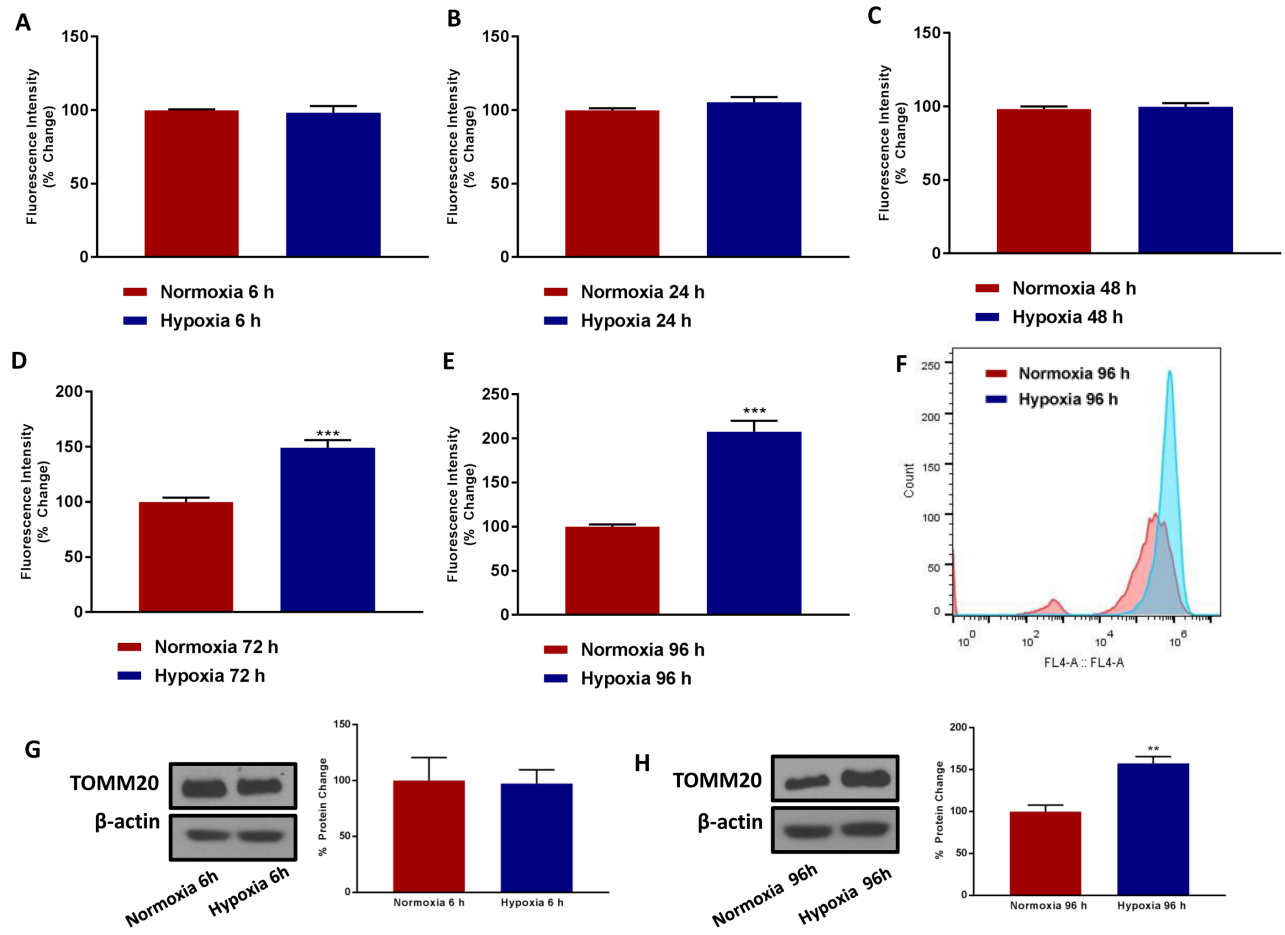
Chronic hypoxia increases mitochondrial mass MCF7 cells were cultured in Normoxia (21% O2) or Hypoxia (1% O2) for 6 h **A.**, 24 h **B.**, 48 h **C.**, 72 h **D.** and 96 h **E.** and then mitochondrial mass was quantitated by FACS analysis using the probe MitoTracker Deep-Red (640-nm). **F.** Representative tracing showing a clear shift to the right for mitochondrial mass in MCF7 cells cultured for 96 h in low oxygen as compared to MCF7 cultured for 96 h in normoxic conditions. Data shown are the mean ± SEM of 3 independent experiments performed in triplicate. (***) *p*<0.001. Evaluation of the mitochondrial protein TOMM20 in MCF7 cells cultured in Normoxia (21 % O2) or Hypoxia (1 % O2) for 6 **G.** and 96 h **H.** Side panels show densitometric analysis of the blots normalized to β-actin. Data shown are the mean ± SEM of 3 independent experiments. (**) *p* < 0.01.

Figure 2 shows that 6, 24 and 48 hours of hypoxia have little or no effect on mitochondrial mass. In contrast, chronic hypoxia increased mitochondrial mass by up to ∼2-fold, at 72 and 96 hours of oxygen-deprivation. A representative FACS tracing is shown in Figure 2F, demonstrating a clear shift to the right. This was confirmed by immuno-blot analysis with TOMM20, an established marker of mitochondrial mass. Therefore, it appears that chronic hypoxia, for 3 to 4 days, preferentially stimulates mitochondrial biogenesis.

We next performed a time course of hypoxia *versus* CSC activity, using the mammosphere assay as a read-out. Initially, MCF7 cell monolayers were cultured under conditions of acute and chronic hypoxia. Then, the cells were trypsinized and re-seeded into low-attachment plates, to detect and quantitatively measure 3D mammosphere forming activity.

**Figure 3:**
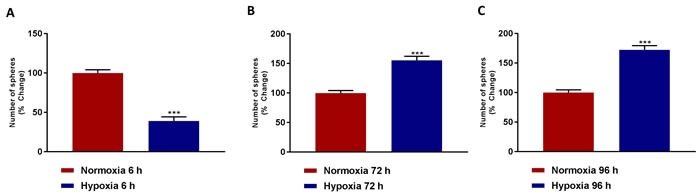
Mammosphere formation is reduced after acute hypoxia and increased after prolonged hypoxia Evaluation of mammosphere formation in MCF7 cells which were cultured in Normoxia (21% O2) or Hypoxia (1% O2) for 6 h **A.**, 72 h **B.** or 96 h **C.** and then seeded in low attachment plate for 5 days before counting. Data shown are the mean ± SEM of 3 independent experiments performed in triplicate. (***) p < 0.001.

Remarkably, Figure 3A shows that acute hypoxia (6 hours) actually inhibits mammosphere formation by >60%. In contrast, Figure 3B, 3C demonstrates that chronic hypoxia (72 and 96 hours) clearly stimulates mammosphere formation, by >1.5-fold. As such, chronic hypoxia appears to drive mitochondrial biogenesis and an increase in cancer stem cell activity, suggesting that these two processes may be functionally linked.

### Doxycycline, an inhibitor of mitochondrial biogenesis, targets and halts the propagation of hypoxia-induced CSC activity

To test the hypothesis that mitochondrial biogenesis is required for hypoxia-induced CSC propagation, we next used the FDA-approved antibiotic Doxycycline. Doxycycline inhibits protein synthesis in bacteria by targeting their ribosomes [6, 7, 9]. However, because of the conserved structural similarities between bacterial and mitochondrial ribosomes, Doxycycline also inhibits mitochondrial biogenesis, as an off-target side-effect in mammalian cells [6, 7, 9].

**Figure 4:**
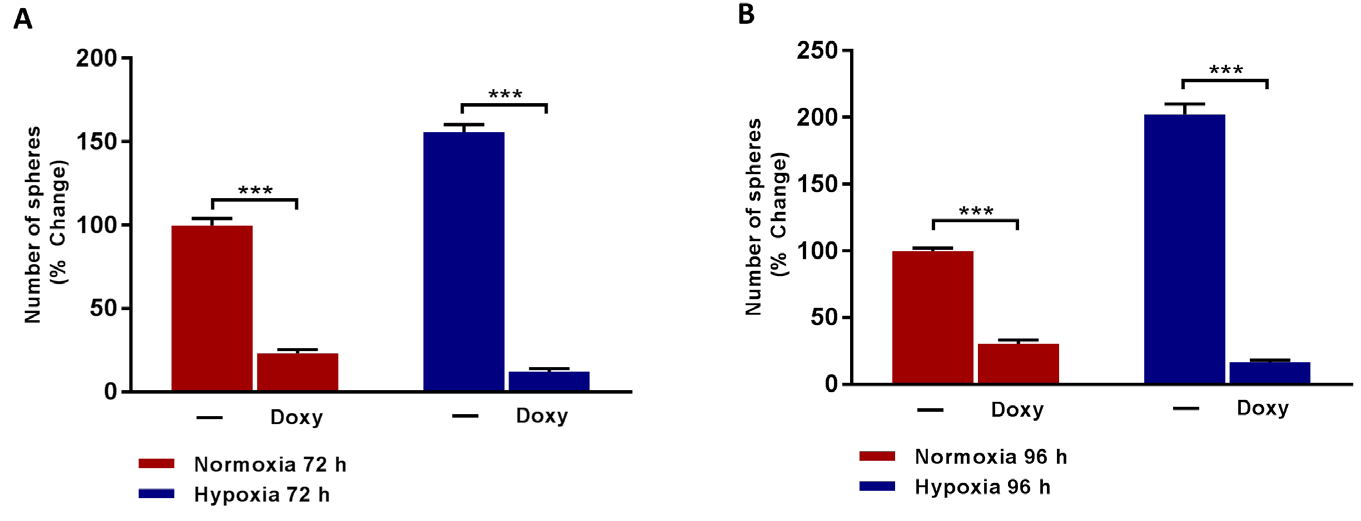
Doxycycline inhibits the formation of mammosphere induced by prolonged hypoxia Evaluation of mammosphere formation in MCF7 cells which were cultured in Normoxia (21% O2) or Hypoxia (1% O2) for 72 h **A.** or 96 h **B.** and then seeded in low attachment plate in the presence of Vehicle (-) or Doxycycline (Doxy, 50 μM) for 5 days before counting. Data shown are the mean ± SEM of 3 independent experiments performed in triplicate. (***) p < 0.001.

Importantly, Figure 4 shows that Doxycycline treatment effectively inhibits hypoxia-induced mammosphere formation, even more effectively than under normoxic conditions. Therefore, Doxycycline is effective after both normoxic and hypoxic pre-treatment conditions, but is actually more effective after chronic hypoxia treatment. Therefore, Doxycycline could be re-purposed to target the propagation of hypoxic CSCs, which are normally strongly resistant to conventional chemotherapy.

### Doxycycline increases the sensitivity of hypoxic CSCs to conventional chemotherapies, such as paclitaxel

We next investigated the implications of our findings for clinical treatments with chemotherapy. Hypoxic CSCs are known to be highly resistant to conventional chemotherapies, such as Paclitaxel [1–4, 12]. We were also able to demonstrate this drug-resistance, in the context of hypoxia.

**Figure 5:**
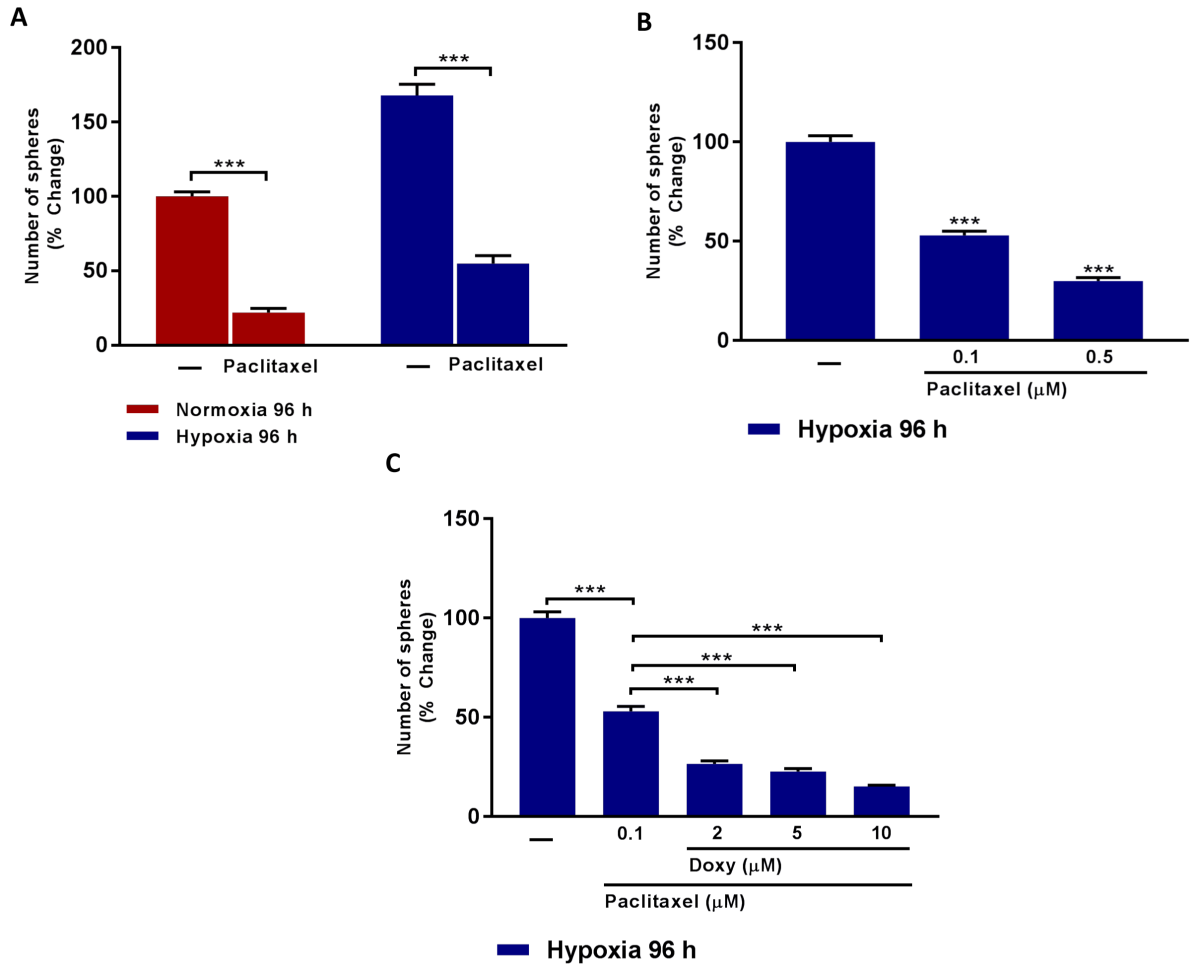
Doxycycline increases hypoxic CSCs sensitivity to paclitaxel treatment **A.** Evaluation of mammosphere formation in MCF7 cells which were cultured in Normoxia (21% O2) or Hypoxia (1% O2) 96 h (A) and then seeded in low attachment plate in the presence of Vehicle (-) or Paclitaxel (0.5 μM) for 5 days before counting. Note that CSCs obtained upon hypoxic conditions are more resistant to Paclitaxel. (***) p < 0.001. **B.** Evaluation of mammosphere formation in MCF7 cells which were cultured in Hypoxia (1% O2) 96 h (B) and then seeded in low attachment plate in the presence of Vehicle (-) or increasing concentrations of Paclitaxel (0.1 to 0.5 μM) for 5 days before counting. Data shown are the mean ± SEM of 3 independent experiments performed in triplicate. (***) p < 0.001. **C.** Evaluation of mammosphere formation in MCF7 cells which were cultured in Hypoxia (1% O2) for 96 h (C) and then seeded in low attachment plate in the presence of Vehicle (-) or Paclitaxel (0.1 μM), alone and in combination with increasing concentration of Doxycycline (2 μM to 10 μM) for 5 days before counting. Note that Doxycycline increases the sensitivity of hypoxic CSCs to paclitaxel treatment. Data shown are the mean ± SEM of 3 independent experiments performed in triplicate. (***) p < 0.001.

Figure 5 directly demonstrates that a significant fraction of CSCs are clearly resistant to treatment with Pactlitaxel and that this chemo-resistance is exacerbated, especially after MCF7 cells are exposed to chronic hypoxia. If we use 0.1 μM Paclitaxel, approximately 50% of the hypoxic CSCs remain Paclitaxel-resistant (Figure 5B). Remarkably, addition of as little as 2 μM Doxycycline removes 50% of the Paclitaxel-resistant CSC activity; similarly, addition of 10 μM Doxycycline inhibits >75% of the Paclitaxel-resistant CSC activity (Figure 5C).

Therefore, we propose that Doxycycline could be used as an add-on to Paclitaxel-treatment, to combat Paclitaxel drug-resistance, normally induced by the hypoxic tumor microenvironment.

### Metabolic phenotyping and proteomic profiling of cancer cells exposed to chronic hypoxia

**Figure 6:**
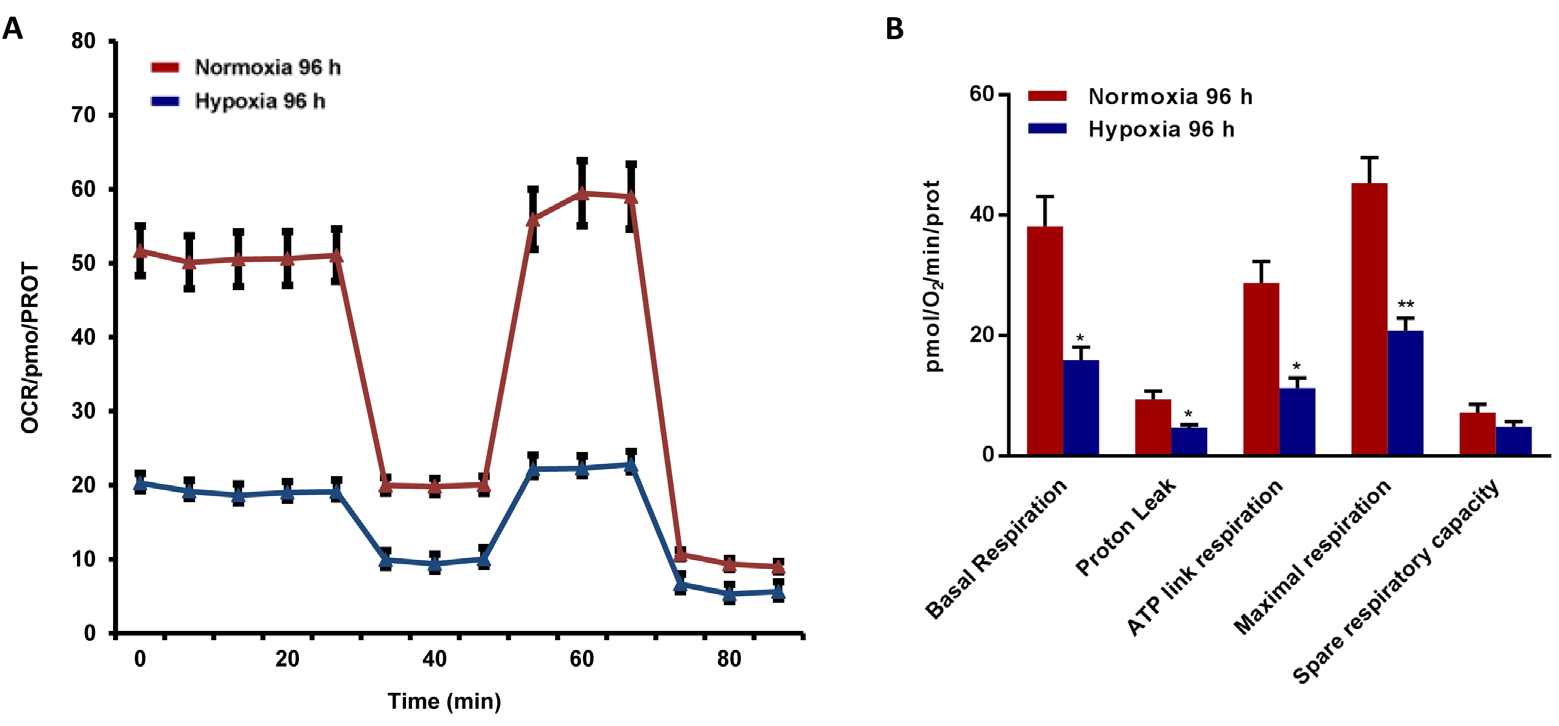
Mitochondrial respiration is inhibited in MCF7 cells exposed to chronic hypoxia The metabolic profile of MCF7 cells cultured in Normoxia (21% O2) or Hypoxia (1% O2) for 96 h, as assessed using the Seahorse XF-e96 analyzer. Representative tracing of metabolic flux. **A.** Significant reduction in basal respiration, proton leak, maximal respiration and ATP levels was observed in MCF7 cells cultured upon Hypoxia vs MCF7 cells cultured upon Normoxia. **B.** Data shown are the mean ± SEM of 3 independent experiments performed in sextuplicate. (*) p < 0.05; (**) p < 0.01.

**Figure 7:**
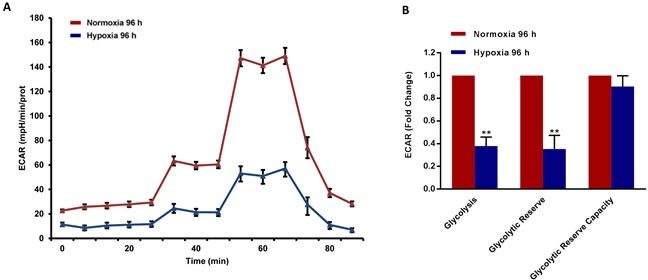
Glycolysis is reduced in MCF7 cells exposed to chronic hypoxia The metabolic profile of MCF7 cells cultured in Normoxia (21% O2) or Hypoxia (1% O2) for 96 h, as assessed using the Seahorse XF-e96 analyzer. Representative tracing of metabolic flux **A.** Significant decrease in glycolysis and glycolytic reserve was observed **B.** Data shown are the mean ± SEM of 3 independent experiments performed in sextuplicate. (**) p < 0.01.

To better assess the metabolic state after chronic hypoxia treatment (96 hours), we next subjected MCF7 cell monolayers to metabolic flux analysis, using the Seahorse XFe96. Interestingly, oxygen-consumption rates (OCR) in normoxia were severely impaired after 96 hours of hypoxic treatment, resulting in a ∼60% reduction in ATP production (Figure 6). Similarly, glycolysis rates, as measured by ECAR (extracellular acidification rate), were also dramatically reduced by >60% (Figure 7). Therefore, MCF7 cells after chronic hypoxia appeared to be in a more quiescent metabolic state.

Consistent with these functional metabolic observations, unbiased proteomics analysis revealed the up-regulation of 45 mitochondrial-related metabolic proteins. This is most likely related to a hypoxia-induced stress response, driving increased mitochondrial biogenesis.

More specifically, Table 1 shows that 3 mitochondrial ribosomal proteins (MRPL4, MRPS35 and MRPL47) were all up-regulated in response to chronic hypoxia. Eleven other proteins related to mitochondrial biogenesis were up-regulated, including: HYOU1, YARS2, NDUFV2, LONP1, POLRMT, COQ9, SARS2, HSPA9, HSPD1, ATP5J, and ATPAF1. Also, a specific inhibitor of mitophagy, namely LRPPRC, which prevents the autophagic digestion of mitochondria, was up-regulated.

**Table 1:** Mitochondrial proteins up-regulated during chronic hypoxia (96 hours) in MCF7 breast cancer cells

Symbol	Description	Fold-Change (Up-regulation)
**HYOU1**	**Hypoxia up-regulated protein 1**	**173.99**
DIABLO	Diablo homolog, mitochondrial	51.91
ECSIT	Evolutionarily conserved signaling intermediate in Toll pathway, mitochondrial	32.26
**MRPL4**	**39S ribosomal protein L4, mitochondrial**	**10.62**
PDK1	[Pyruvate dehydrogenase (acetyl-transferring)] kinase isozyme 1, mitochondrial	8.39
HIBADH	3-hydroxyisobutyrate dehydrogenase, mitochondrial	8.28
**YARS2**	**Tyrosine--tRNA ligase, mitochondrial**	**7.58**
AK4	Adenylate kinase 4, mitochondrial	7.07
**NDUFV2**	**NADH dehydrogenase [ubiquinone] flavoprotein 2, mitochondrial**	**6.94**
ALDH6A1	Methylmalonate-semialdehyde dehydrogenase [acylating], mitochondrial	6.17
MICU1	Calcium uptake protein 1, mitochondrial	5.10
**LONP1**	**Lon protease homolog, mitochondrial**	**4.36**
ACADSB	Short/branched chain specific acyl-CoA dehydrogenase, mitochondrial	4.25
MAVS	Mitochondrial antiviral-signaling protein	4.12
SLC25A11	Mitochondrial 2-oxoglutarate/malate carrier protein	3.39
**LRPPRC**	**Leucine-rich PPR motif-containing protein, mitochondrial**	**2.92**
MTHFD2	Bifunctional methylenetetrahydrofolate dehydrogenase/cyclohydrolase, mitochondrial	2.63
**MRPS35**	**28S ribosomal protein S35, mitochondrial**	**2.58**
ACAD9	Acyl-CoA dehydrogenase family member 9, mitochondrial	2.33
ABAT	4-aminobutyrate aminotransferase, mitochondrial	2.28
SLC25A13	Calcium-binding mitochondrial carrier protein Aralar2	2.21
ACADVL	Very long-chain specific acyl-CoA dehydrogenase, mitochondrial	2.21
**POLRMT**	**DNA-directed RNA polymerase, mitochondrial**	**2.18**
HADH	Hydroxyacyl-coenzyme A dehydrogenase, mitochondrial	2.17
**COQ9**	**Ubiquinone biosynthesis protein COQ9, mitochondrial**	**2.12**
**SARS2**	**Serine--tRNA ligase, mitochondrial**	**2.12**
**HSPA9**	**Stress-70 protein, mitochondrial**	**2.08**
CS	Citrate synthase, mitochondrial	2.08
PCCB	Propionyl-CoA carboxylase beta chain, mitochondrial	2.05
**HSPD1**	**60 kDa heat shock protein, mitochondrial**	**2.01**
DECR1	2,4-dienoyl-CoA reductase, mitochondrial	2.01
ACOT9	Acyl-coenzyme A thioesterase 9, mitochondrial	1.97
GLS	Glutaminase kidney isoform, mitochondrial	1.97
ACADM	Medium-chain specific acyl-CoA dehydrogenase, mitochondrial	1.95
**ATP5J**	**ATP synthase-coupling factor 6, mitochondrial**	**1.93**
ACSM2B	Acyl-coenzyme A synthetase ACSM2B, mitochondrial	1.86
MMAB	Cob(I)yrinic acid a,c-diamide adenosyltransferase, mitochondrial	1.86
CPOX	Oxygen-dependent coproporphyrinogen-III oxidase, mitochondrial	1.86
SUCLG2	Succinyl-CoA ligase [GDP-forming] subunit beta, mitochondrial	1.84
**MRPL47**	**39S ribosomal protein L47, mitochondrial**	**1.84**
CPT2	Carnitine O-palmitoyltransferase 2, mitochondrial	1.79
IDH3B	Isocitrate dehydrogenase [NAD] subunit beta, mitochondrial	1.76
SLC25A24	Calcium-binding mitochondrial carrier protein SCaMC-1	1.74
**ATPAF1**	**ATP synthase mitochondrial F1 complex assembly factor 1**	**1.74**
NNT	NAD(P) transhydrogenase, mitochondrial	1.73

Note that 3 mitochondrial ribosomal proteins **(MRPL4, MRPS35 and MRPL47)** were all up-regulated in response to chronic hypoxia. Other proteins related to mitochondrial biogenesis were up-regulated, including: **HYOU1, YARS2, NDUFV2, LONP1, POLRMT, COQ9, SARS2, HSPA9, HSPD1, ATP5J,** and **ATPAF1**. A specific inhibitor of mitophagy, namely **LRPPRC,** which prevents the autophagic digestion of mitochondria, was also up-regulated.

Interestingly, HYOU1 (Hypoxia up-regulated protein 1) was over-expressed by >170-fold. Importantly, HYOU1 (a.k.a., Orp150) is a mitochondrial chaperone protein that belongs to the heat shock protein 70 family, which is known to be involved in mitochondrial protein folding and confers cyto-protection under hypoxic conditions [13, 14]. In addition, other proteins that are part of the OXPHOS complexes were also up-regulated, such as NDUFV2, which was increased by nearly 7-fold.

### Chronic hypoxia induces CSC markers, such as ALDH

**Figure 8:**
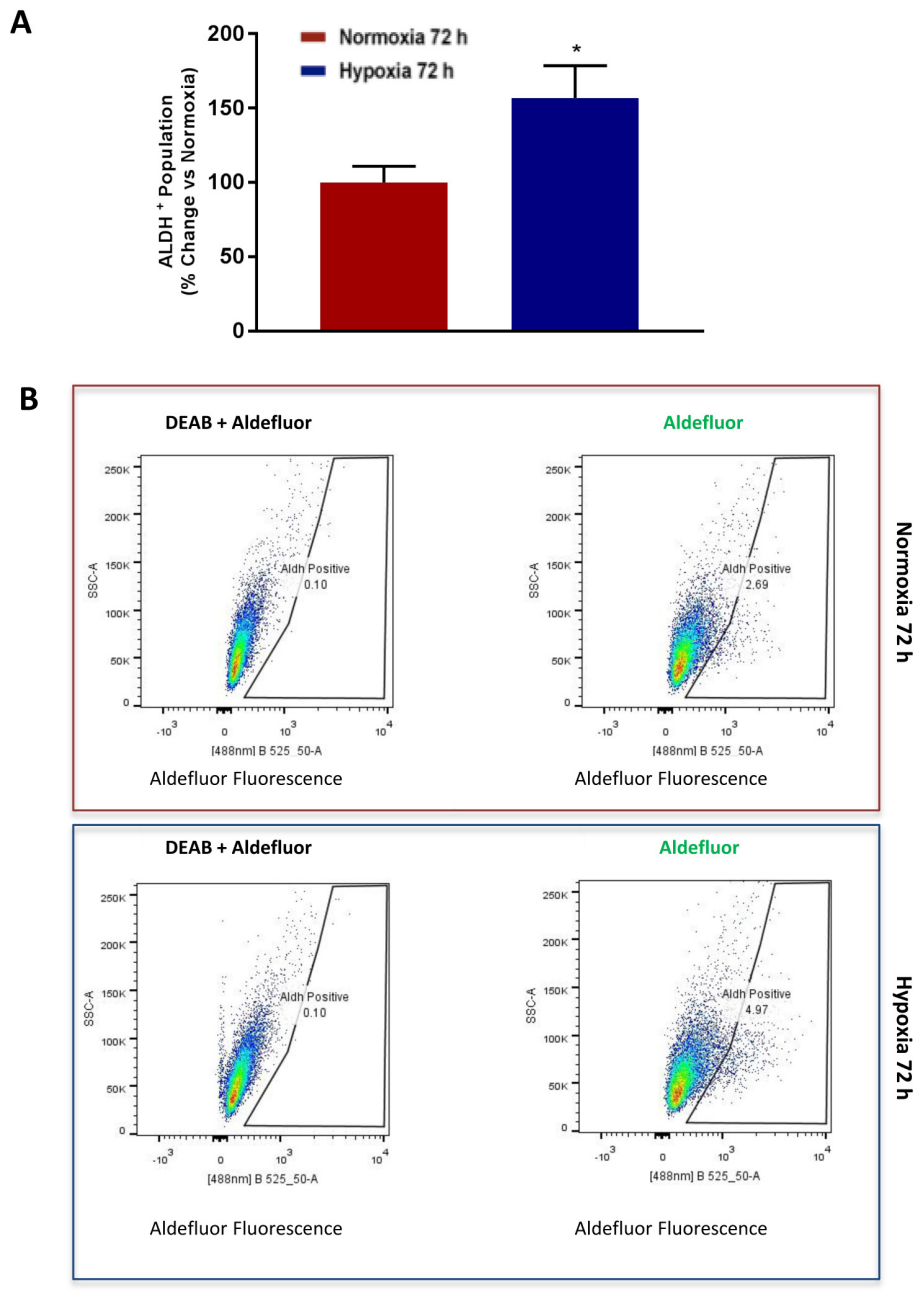
Chronic hypoxia increases the CSC marker ALDH Evaluation of ALDEFLUOR activity, an independent marker of CSCs in MCF7 cells cultured in Normoxia (21% O2) or Hypoxia (1% O2) for 72 h **A.**. Each sample was normalized using diethylaminobenzaldehyde (DEAB), a specific ALDH inhibitor, as negative control. The tracing of representative samples is shown **B.** Data shown are the mean ± SEM of 3 independent experiments performed in triplicate. (*) p < 0.05.

ALDH activity is now a well-established CSC marker for detecting and enriching CSC activity by flow-cytometry [15–17]. ALDH activity can be measured by FACS, analysis using the Aldefluor fluorescent probe [15–17]. Consistent with our current observations that chronic hypoxia increases mammosphere formation by >1.5-fold, we also observed a >1.5-fold increase in the number of ALDH(+) cells by FACS (Figure 8).

### Chronic hypoxia induces oxidative stress: Mito-TEMPO, a mitochondrial anti-oxidant, halts mammosphere formation

**Figure 9:**
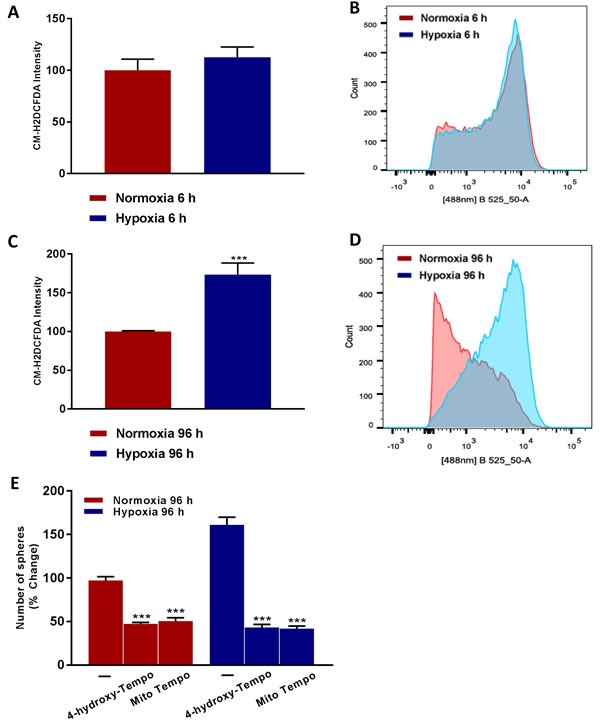
Higher ROS levels ‘boost’ hypoxic CSC propagation Evaluation of ROS levels by FACS analysis using the CM-H2DCFDA probe in MCF7 cells cultured in Normoxia (21% O2) or Hypoxia (1% O2) for 6 h **A.** and 96 h **C.** Note that prolonged hypoxia significantly increases ROS levels. **B.**, **D.** Representative tracing showing a clear shift to the right for mitochondrial mass in MCF7 cells cultured for 96 h in low oxygen as compared to MCF7 cultured for 96 h in normoxic conditions. Mammosphere formation is inhibited in MCF7 cells cultured in Normoxia (21% O2) or Hypoxia (1% O2) for 96 h **E.** in the presence of Vehicle (-) and the mitochondria-targeted superoxide scavenger Mito-TEMPO (100 μM) or 4-Hydroxy-TEMPO (100 μM) (C). Data shown are the mean ± SEM of 3 independent experiments performed in triplicate. (***) p < 0.001.

To better understand the mechanism(s) underpinning how chronic hypoxia drives an increase in cancer stem cell activity, we hypothesized that oxidative stress might be the ‘root cause’. To test this hypothesis directly, we quantitatively measured ROS production after acute hypoxia (6 hours) and after chronic hypoxia (96 hours), using the probe CM-H2DCFDA by FACS analysis. Interestingly, Figure 9A-9D shows that chronic hypoxia induces a >1.5-fold increase in ROS production, but that there is no increase in ROS production after acute hypoxia.

To determine if oxidative stress drives the observed hypoxia-induced increase in ‘stemness’, we also examined if simple anti-oxidants can inhibit mammosphere formation. For this purpose, we used TEMPO-derivatives that behave as membrane-permeable SOD-mimetic agents, which scavenge superoxide anions and other free radicals. Figure 9E demonstrates that both i) 4-hydroxy-TEMPO and ii) Mito-TEMPO effectively inhibited mammosphere formation by >70%, at a concentration of 100 μM. Importantly, Mito-TEMPO is a mitochondrially-targeted form of TEMPO, which contains a chemical mitochondrial targeting signal [8]. Therefore, we conclude that mitochondrial oxidative stress appears to be one of the key underlying causes of hypoxia-induced ‘stemness’.

### Chronic hypoxia activates p44/42-MAPK signalling, without modifying HIF-1α expression

HIF1-alpha is a well-known transcriptional mediator of the acute effects of hypoxia, but its functional role in chronic hypoxia is less well defined [18–22]. Therefore, we monitored the expression levels of HIF1-alpha in this context, by immunoblot analysis. We also measured p-ERK-1/2 activation, using phospho-specific antibody probes, for comparison purposes.

**Figure 10:**
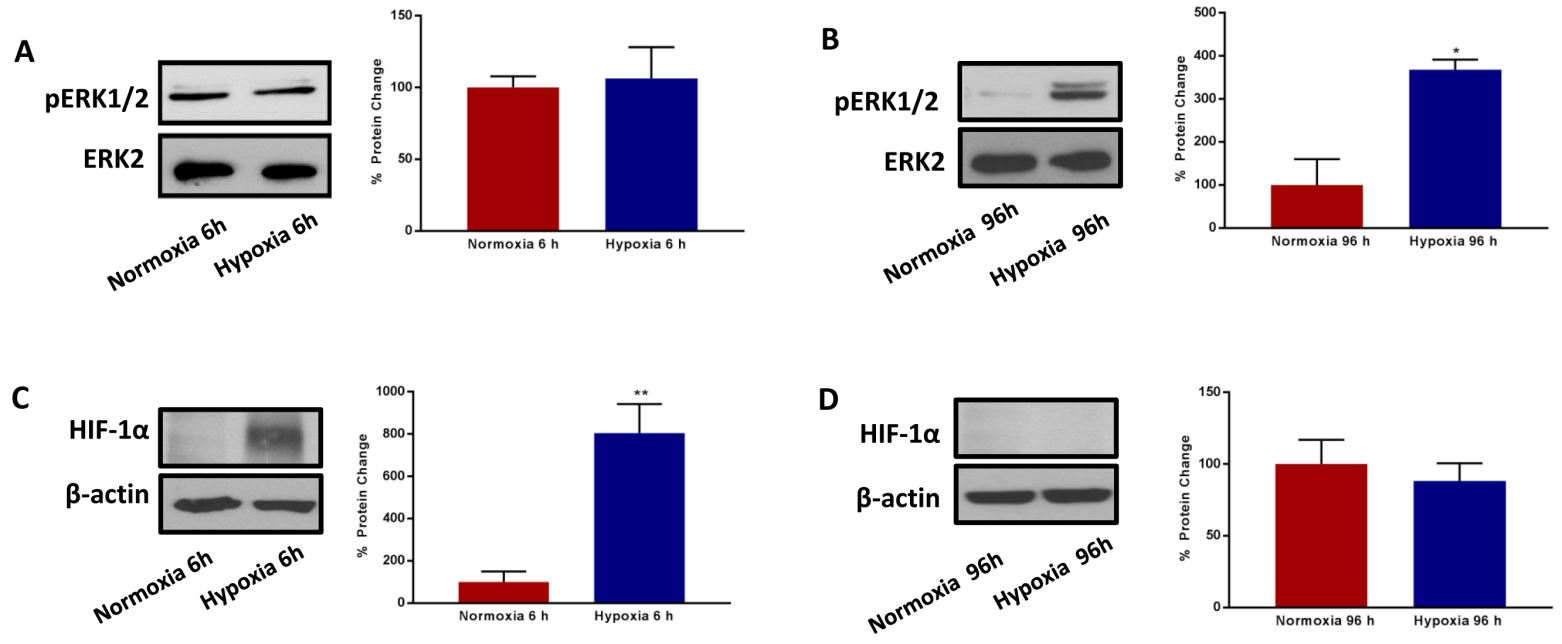
Prolonged hypoxia activates MAPK signalling, without modifying HIF-1α expression Evaluation of ERK1/2 phosphorylation in MCF7 cells cultured in Normoxia (21% O2) or Hypoxia (1% O2) for 6h **A.** and 96 h **B.** Side panels show densitometric analysis of the blots normalized to ERK2. Evaluation of HIF-1α expression in MCF7 cells cultured in Normoxia (21% O2) or Hypoxia (1% O2) for 6 h **C.** and 96 h **D.** Side panels show densitometric analysis of the blots normalized to β-actin. Data shown are the mean ± SEM of 3 independent experiments. (*) p < 0.05; (**) p < 0.01.

Interestingly, Figure 10 shows that HIF1-alpha is strongly upregulated during acute hypoxia, as expected, but it remains undetectable during chronic hypoxia. Conversely, the levels of activated phospho-ERK-1/2 were unchanged by acute hypoxia, but were significantly elevated by chronic hypoxia. Thus, these two very different signalling molecules may contribute to metabolic signalling at different phases of the hypoxia-induced stress response. The activation of ERK-1/2 by chronic hypoxia may provide a key stimulus for enhancing anchorage-independent growth.

### Investigating the role of fatty acid oxidation (FAO) in mitochondrial biogenesis and CSC propagation

Fatty acid oxidation (FAO) is the process by which fatty acids are catabolized in mitochondria and peroxisomes, to generate Acetyl-CoA, which can then enter the TCA/Krebs cycle. In this process, the energy generated for each Acetyl-CoA molecule oxidized, results in 1 GTP and 11 ATP molecules.

Further bioinformatics analysis of the high-resolution proteomics data presented in Table 1 reveals that a significant number of metabolic enzymes related to mitochondrial FAO are up-regulated during chronic hypoxia. More specifically, twelve mitochondrial proteins involved in FAO were induced by chronic hypoxia, including: HIBADH, ACADSB, ACAD9, ACADVL, HADH, PCCB, DECR1, ACOT9, ACADM, ACSM2B, SUCLG2 and CPT2. This is summarized in more detail in Table 2.

**Table 2:** Mitochondrial proteins up-regulated during chronic hypoxia: focus on fatty acid oxidation (FAO)

Symbol	Description	Fold-Change (Up-regulation)
HIBADH	3-hydroxyisobutyrate dehydrogenase, mitochondrial	8.28
ACADSB	Short/branched chain specific acyl-CoA dehydrogenase, mitochondrial	4.25
ACAD9	Acyl-CoA dehydrogenase family member 9, mitochondrial	2.33
ACADVL	Very long-chain specific acyl-CoA dehydrogenase, mitochondrial	2.21
HADH	Hydroxyacyl-coenzyme A dehydrogenase, mitochondrial	2.17
PCCB	Propionyl-CoA carboxylase beta chain, mitochondrial	2.05
DECR1	2,4-dienoyl-CoA reductase, mitochondrial	2.01
ACOT9	Acyl-coenzyme A thioesterase 9, mitochondrial	1.97
ACADM	Medium-chain specific acyl-CoA dehydrogenase, mitochondrial	1.95
ACSM2B	Acyl-coenzyme A synthetase ACSM2B, mitochondrial	1.86
SUCLG2	Succinyl-CoA ligase [GDP-forming] subunit beta, mitochondrial	1.84
CPT2	Carnitine O-palmitoyltransferase 2, mitochondrial	1.79

These results suggest that FAO may be intimately related to mitochondrial biogenesis and CSC propagation. To test this hypothesis independently of hypoxia, we next used another distinct, more direct, stimulus to drive increased FAO and mitochondrial biogenesis. For this purpose, MCF7 cells were treated with Valproic acid, an FDA-approved drug commonly used for the treatment of epilepsy. In this context, Valproic acid is thought to behave as a fatty acid, stimulating FAO. In fact, Valproic acid is chemically-classified as a branched short-chain fatty acid.

**Figure 11:**
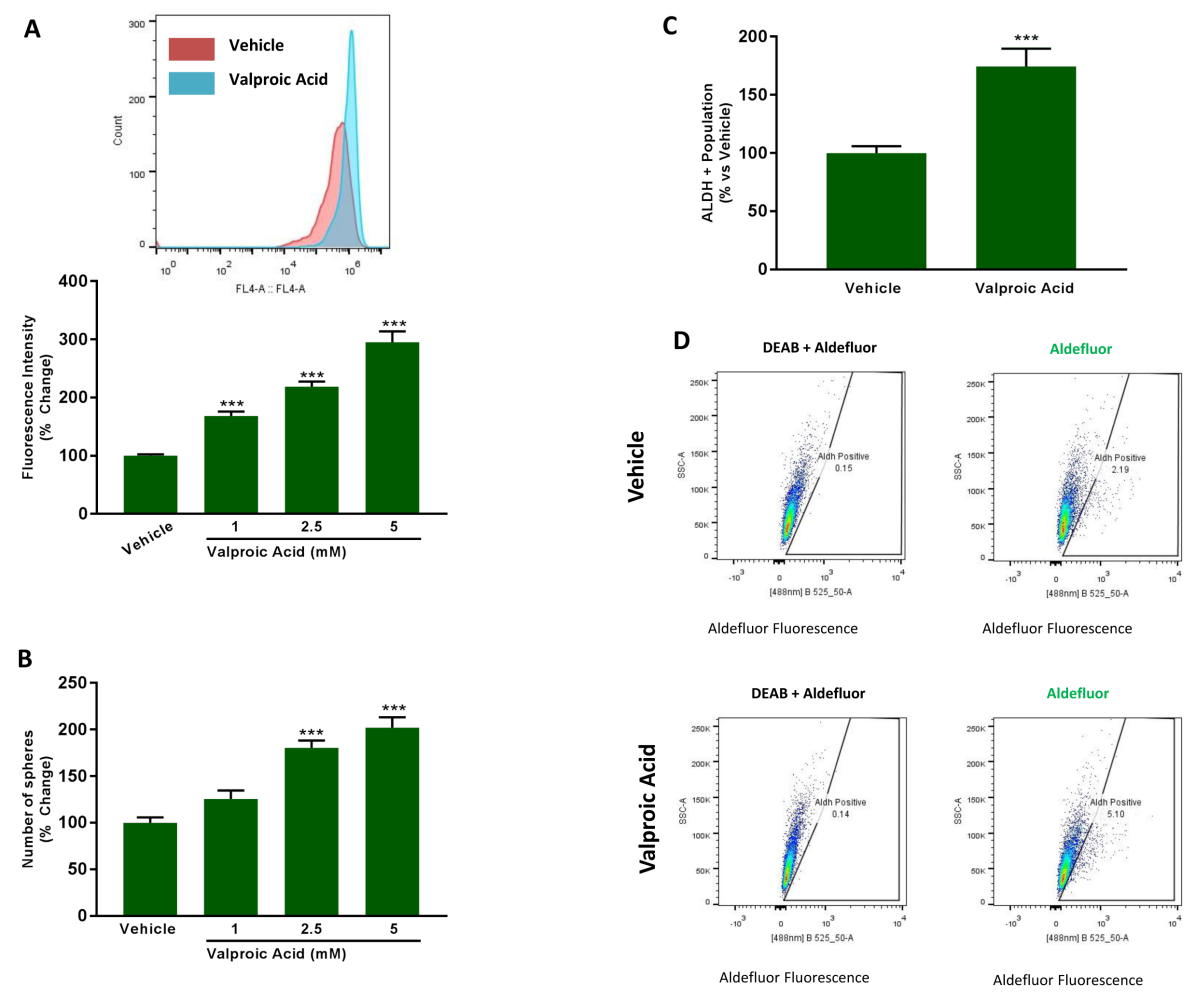
Valproic acid increases mitochondrial mass and induces ‘stemness’ features **A.** Mitochondrial mass. MCF7 cells were treated for 72 h with Vehicle or increasing concentrations of Valproic Acid (1 to 5 mM). Then, mitochondrial mass was evaluated by FACS analysis using the probe MitoTracker Deep-Red (640-nm). Inset displays a representative tracing showing a clear shift to the right for mitochondrial mass in MCF7 cells treated with 2.5 mM Valproic Acid vs MCF7 treated with Vehicle. **B.** Mammosphere formation. MCF7 cells which were treated with Vehicle or increasing concentrations of Valproic Acid (1 to 5 mM) for 72 h. Then, treatments were removed and cells were incubated in regular medium for additional 72 h before seeding in low attachment plate for 5 days in the presence of treatments. **C.** ALDEFLUOR activity, an independent marker of CSCs. MCF7 cells were treated with vehicle and Valproic Acid (2.5 mM) for 72 h. Each sample was normalized using diethylaminobenzaldehyde (DEAB), a specific ALDH inhibitor, as negative control. The tracing of representative samples is shown **D.** In panels A-C, data shown are the mean ± SEM of 3 independent experiments performed in triplicate. (***) p < 0.001.

Figure 11A shows that treatment with increasing concentrations of Valproic acid (0, 1, 2.5 and 5 mM) is indeed sufficient to stimulate mitochondrial biogenesis, resulting in an up to 3-fold increase in mitochondrial mass. Similarly, addition of Valproic acid increased mammosphere formation, by up to 2-fold (Figure 11B). As the effects we observed were near maximal at 2.5 mM and some toxicity was observed at 5 mM, we decided to perform all subsequent experiments with 2.5 mM Valproic acid. At 2.5 mM, Valproic acid increased ALDH activity by >1.5-fold, consistent with an increase in ‘stemness’ (Figure 11C, 11D).

To validate the idea that Valproic acid increased CSC propagation by a metabolic mechanism, we used two distinct inhibitors of FAO, namely Etomoxir and Perhexiline, both of which specifically target the enzyme CPT (carnitine O-palmitoyltransferase). Interestingly, Perhexiline is used clinically in New Zealand and Australia, as a preventative treatment for ischemic heart disease.

**Figure 12:**
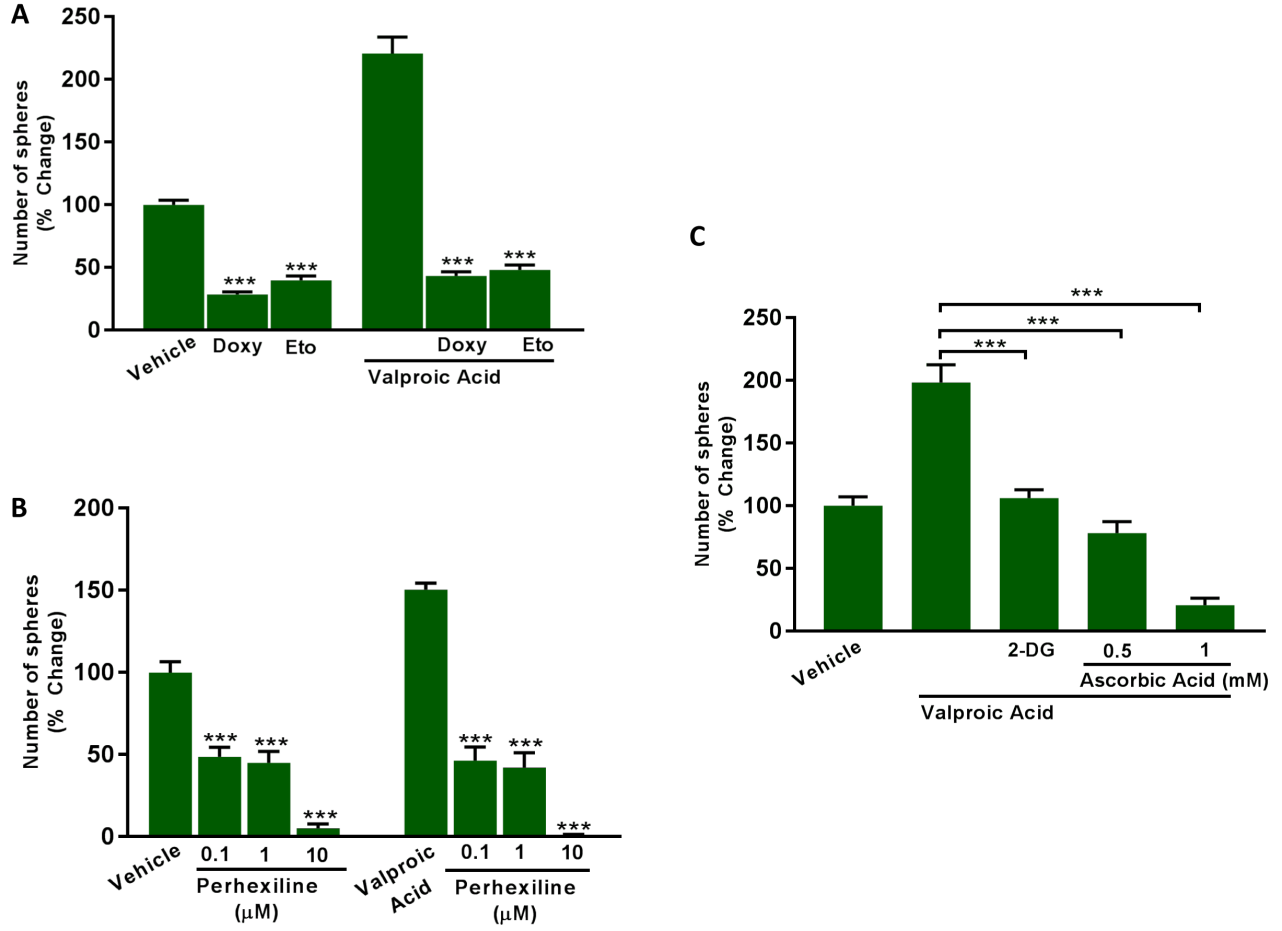
Inhibitors of fatty acid oxidation (FAO), mitochondrial biogenesis and glycolysis all block Valproic acid driven CSC propagation **A.**, **B.** Evaluation of mammosphere formation in MCF7 cells which were treated with Vehicle or Valproic Acid (2.5 mM) for 72 h. Then, the cells were allowed to recover in regular medium for additional 72 h and subsequently seeded in low attachment plate for 5 days in the presence of Valproic Acid, alone and in combination with the mitochondrial inhibitor (A) Doxycycline (50 μM), or the carnitine palmitoyltransferase-1 inhibitors (A) Etomoxir (200 μM), and (B) Perhexiline (0.1 to 10 μM). **C.** Evaluation of mammosphere formation in MCF7 cells which were treated with Vehicle or Valproic Acid (2.5 mM) for 72 h. Then, the cells were allowed to recover in regular medium for additional 72 h and subsequently seeded in low attachment plate for 5 days in the presence of Valproic Acid, alone and in combination with the glycolysis inhibitors 2-Deoxy-glucose (2-DG, 10 mM) and Ascorbic Acid (0.5 to 1 mM). Data shown are the mean ± SEM of 3 independent experiments performed in triplicate. (***) p < 0.001.

Figure 12A, 12B shows that Etomoxir (200 μM) and Perhexiline (0.1, 1 and 10 μM) effectively inhibit both basal and Valproic acid augmented CSC propagation. Similar results were also obtained with Doxycycline (50 μM), which functions to inhibit mitochondrial biogenesis (Figure 12A).

Glycolysis is normally required to provide additional TCA cycle intermediates for the mitochondrial processing of Acetyl-CoA. Consistent with this idea, treatment with glycolysis inhibitors (2-DG or Vitamin C (ascorbic acid)) was also sufficient to inhibit Valproic acid augmented CSC propagation (Figure 12C). Under these conditions the IC-50 for 2-DG was 10 mM, while the IC-50 for Vitamin C was ∼0.5 mM. If we compare with our previously published results under basal conditions [10], Vitamin C was 2X as potent under Valproic acid augmented conditions.

In summary, it appears that both mitochondrial biogenesis and CSC propagation are metabolically linked to FAO, which can be functionally stimulated by Valproic acid (an FDA-approved drug) and inhibited by CPT inhibitors, such as Etomoxir and Perhexiline. Interestingly, the use of doxycycline or glycolysis inhibitors (2-DG and Vitamin C) was also sufficient to override the stimulatory effects of Valproic acid on CSC propagation.

## DISCUSSION

### Repositioning the FDA-approved antibiotic Doxycycline to target hypoxic CSCs

**Figure 13:**
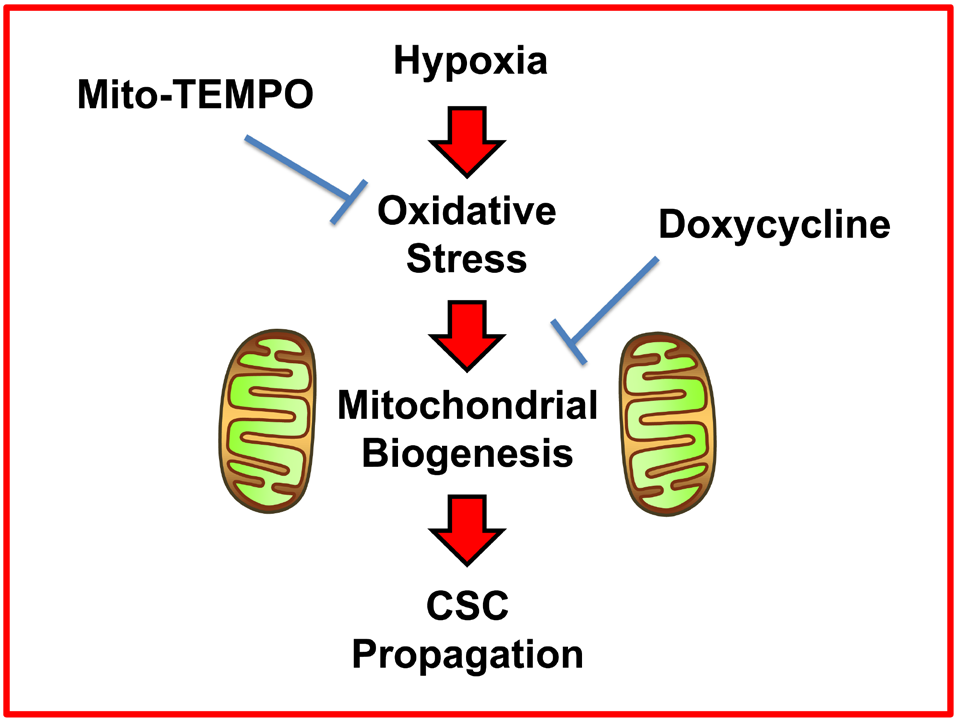
Schematic diagram summarizing the role of ROS production, oxidative stress and mitochondrial biogenesis in maintaining “stemness” in cancer cells Here, we show that the propagation of hypoxic CSCs can be effectively targeted and halted with i) a mitochondrial anti-oxidant (Mito-TEMPO) or ii) an inhibitor of mitochondrial biogenesis (Doxycycline).

Here, we report a new mechanism underlying how prolonged hypoxia drives the onset of enhanced stem cell characteristics in cancer cells. This prolonged or chronic hypoxia leads to elevated ROS production and oxidative stress, which in turn drives increased mitochondrial biogenesis as a stress response. More specifically, this hypoxic stress response fosters an increase in overall mitochondrial mass in CSCs. Based on these new mechanistic observations, we next employed two well-established small molecules to directly target oxidative stress and mitochondrial protein synthesis, in hypoxic CSCs. As a consequence, we now demonstrate that i) Mito-TEMPO (a mitochondrial anti-oxidant) [8] and ii) Doxycycline (an antibiotic that inhibits mitochondrial protein translation) [9–11] can both be used to functionally target hypoxic CSCs (summarized in Figure 13).

Therefore, we propose that Doxycycline is a non-toxic FDA-approved antibiotic that could be re-positioned to specifically eradicate hypoxic CSCs. We envision that Doxycycline would be used alone or in combination with other chemotherapy agents, such as Paclitaxel. In fact, we show here that Doxycycline can be used to target the Paclitaxel-resistant sub-population of hypoxic CSCs.

### Implications of Doxycycline for combating anti-angiogenic therapy resistance

Over the last decade, anti-angiogenic therapies have emerged as promising anti-cancer agents, based on their ability to target tumor blood vessels, depriving cancer cells of essential nutrients [23–26]. However, clinical and pre-clinical data now questions the long-term benefits of anti-angiogenic therapies. For example, administration of anti-angiogenic agents has been shown to actually increase tumor invasiveness and metastasis [23–26]. The mechanistic explanation for the failure of angiogenesis inhibitors is their ability to generate intra-tumoral hypoxia, which then stimulates CSC survival and propagation [23–26].

Indeed, environmental stressors like chronic hypoxia activate a complex response, that includes the stimulation of an “emergency” biochemical and biological programs. Under these conditions, cancer cells survive by entering into a transient quiescent state, which is reversed when appropriate environmental conditions are sufficient to support cell proliferation and ultimately metastasis [27]. As such, the effectiveness of anti-angiogenic agents could be improved by using combination strategies aimed at inhibiting both cancer- and cancer stem-like cells. Based on our current observations, we suggest the combined use of Doxycycline, with angiogenesis inhibitors, such as Bevacizumab (Avastin). This new proposed combination therapy would effectively block both: i) blood vessel formation and ii) CSC propagation, ultimately making anti-angiogenic therapy more effective.

Additionally, our data indicate that Doxycycline’s inhibitory action on CSC propagation is particularly efficient after a hypoxic stress as compared with normoxic conditions, suggesting that anti-angiogenic agents, by generating intratumoral hypoxia, could sensitize the dormant CSCs to the inhibitory effects of mitochondria targeting agents. Although further validation of this therapeutic strategy is needed, the use of mitochondrial biogenesis inhibitors could ultimately allow anti-angiogenic drugs to fulfill their therapeutic potential.

In order to eradicate hypoxic cancer cells, many other investigators have sought to generate novel therapeutics that specifically interfere with HIF1-alpha function, the major hypoxia-induced transcription factor [18, 19]. However, based on our current findings, HIF1-apha is preferentially expressed only during the acute phase of hypoxia (6 hours), but is virtually undetectable or completely absent, under conditions of chronic hypoxia (96 hours). Instead, we find that phospho-ERK-1/2 is hyper-activated during chronic hypoxia, but not during acute hypoxia, showing just the opposite activation pattern as HIF1-apha. Based on these findings, we conclude that HIF1-apha inhibitors may not be that clinically relevant for the targeting of hypoxic CSCs, especially under chronic conditions, since the actions of HIF1-alpha appear to be largely confined to acute hypoxia.

### Linking mitochondrial biogenesis to CSC propagation and asymmetric cell division

During our hypoxia experiments, we observed that MCF7 cells subjected to chronic hypoxia (for 96 hours) have dramatically reduced metabolic activity, characterized by very low levels of both i) mitochondrial oxygen consumption and ii) glycolysis; this finding is suggestive of a more quiescent metabolic state. In contrast, chronically hypoxic MCF7 cells showed marked increases in mitochondrial mass, a surrogate marker of mitochondrial biogenesis. Therefore, increased mitochondrial biogenesis may be a primary driver of increased “stemness”, rather than augmented cell metabolism.

In direct support of this ‘working hypothesis’, Sabatini, Weinberg and colleagues have shown that new mitochondrial biogenesis is absolutely required for asymmetric cell division in CSCs [28]. More specifically, they observed that during asymmetric cell division, new CSCs retain the newly-generated mitochondria, while the old mitochondria are transferred during cytokinesis to the non-stem cells (or daughter cells) [28]. Therefore, inhibition of mitochondrial biogenesis should block asymmetric cell division, reducing “stemness”. This mechanistic interpretation would directly explain why Doxycycline is so effective at halting mammosphere formation, which is strictly dependent on asymmetric cell division in CSCs.

### Dissecting the functional role of FAO in mitochondrial biogenesis and CSC propagation

Since we observed that hypoxia specifically induced >10 mitochondrial enzymes associated with FAO (Table 2), we used Valproic acid as an independent and experimentally convenient means to activate FAO.

Valproic acid (2-propylpentanoic acid) is a simple fatty acid, which functions as a substrate for fatty acid beta-oxidation (FAO) [29–32]. Valproic acid is completely metabolized by mitochondria, indicating that it can directly function as a mitochondrial fuel for FAO [28–32]. This FDA-approved drug is widely-used clinically for the management of patients with epilepsy.

Although Valproic acid has been shown to elicit anti-proliferative and anti-apoptotic actions in diverse cancer cell systems [33–34], its actual effectiveness as an anti-cancer agent is still a controversial and hotly-debated topic, as suggested by several recent clinical trials [35–37]. On the other hand, Valproic acid has been shown to increase the CSC population in glioblastoma cells and to “reprogram” differentiated triple-negative breast cancer cells to become quiescent stem-like cancer cells [38–39]. Supporting these studies, an increase in cell migration, invasion and a switch towards the epithelial-mesenchymal transition (EMT) phenotype has been detected, in various cancer cells treated with Valproic acid [40–41].

**Figure 14:**
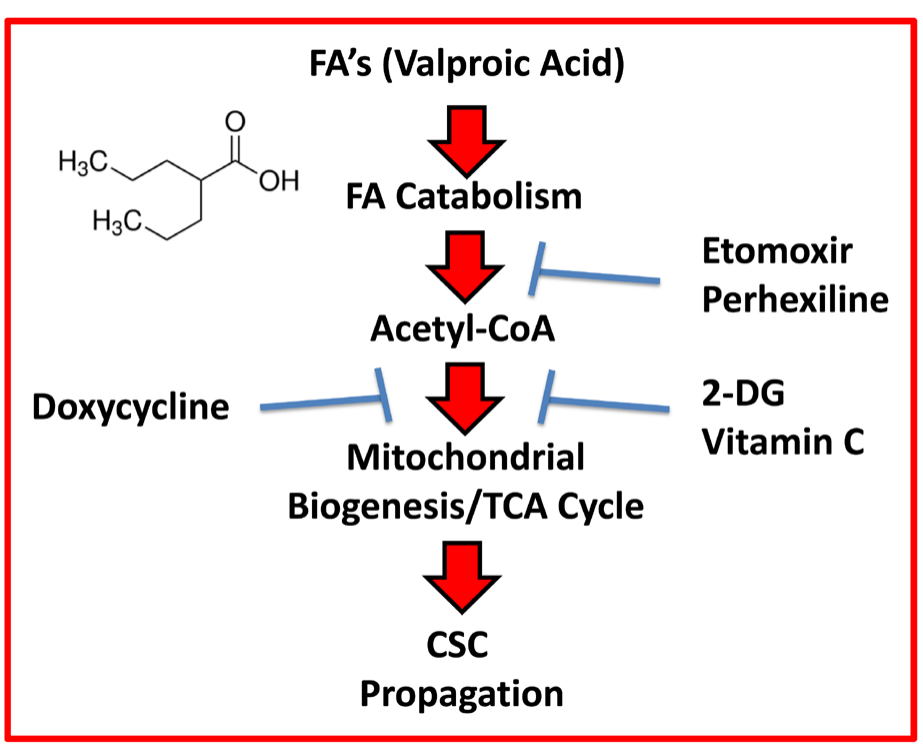
Schematic diagram summarizing the role of fatty acid oxidation (FAO) in driving CSC propagation Here, we show that mitochondrial biogenesis and the propagation of CSCs can be stimulated by Valproic acid (VA, an FDA-approved drug), which is a branched short-chain fatty acid. Moreover, we demonstrate that VA-induced CSC propagation can be blocked by inhibitors of i) FAO (Etomoxir and Perhexiline), ii) mitochondrial biogenesis (Doxycycline) and/or iii) glycolysis (2-DG and Vitamin C). Importantly, Perhexiline and Doxycycline are used clinically for other medical indications, so they could be re-purposed. Vitamin C is a micronutrient that is also readily available (over-the-counter) and can be used safely, at relatively high oral or i.v. dosages.

Interestingly, we observed here that treatment of MCF7 cells with Valproic acid was indeed sufficient to increase both i) mitochondrial biogenesis and ii) CSC propagation. The Valproic acid induced increase in CSC propagation that we observed could be halted by using well-established inhibitors of FAO, namely Etomoxir and Perhexiline, that target the enzyme CPT (summarized in Figure 14). Importantly, Etomoxir and Perhexiline also inhibited basal CSC propagation, that was not induced by Valproic acid.

Therefore, based on the above experimental evidence, we believe that FAO is also a critical source of metabolic energy for fueling the propagation of CSCs. Perhexiline is used clinically, in New Zealand and Australia, as a prophylactic anti-anginal agent. As such, Perhexiline could be re-purposed to inhibit FAO in CSCs. Similarly, treatment with Doxycycline, another FDA-approved drug, was sufficient to combat the stimulatory effects of Valproic acid on CSC propagation.

## CONCLUSIONS

In summary, we provide new functional evidence that Doxycycline-mediated inhibition of mitochondrial biogenesis may indeed be sufficient to eliminate hypoxic CSCs. Based on these new findings, we believe that future Phase II clinical trials may be warranted, to re-purpose Doxycycline as an anti-cancer agent targeting chronic hypoxia.

## MATERIALS AND METHODS

### Materials

Doxycycline, Paclitaxel and Perhexiline Maleate were dissolved in DMSO. A 0.01 M solution of paclitaxel in DMSO was further diluted with culture medium. Mito Tempo, 4-Hydroxy-Tempo and Etomoxir were dissolved in H2O. 2-deoxy-D-glucose (2-DG) and Ascorbic Acid were dissolved in culture medium. All compounds were from Sigma Aldrich.

### Cell cultures

MCF7 breast cancer cells were obtained from ATCC and cultured in DMEM (Sigma Aldrich). For hypoxic stimulation MCF7 cells were cultured in low glucose DMEM (Sigma Aldrich) in a multi-gas N2/CO2 hypoxic chamber at 1 % pO2; parallel, MCF7 cells were cultured in low glucose DMEM at 21 % O2 to serve as a normoxic control.

### Mammosphere formation

A single cell suspension of MCF7 cells previously exposed to Normoxia (21% O2) or Hypoxia (1% O2) for 6h, 72h or 96h was prepared using enzymatic (1x Trypsin-EDTA, Sigma Aldrich), and manual disaggregation (25 gauge needle) [42]. Cells were then plated at a density of 500 cells/cm2 in mammosphere medium (DMEM-F12/ B27 / 20-ng/ml EGF/PenStrep) in nonadherent conditions, in culture dishes coated with (2-hydroxyethylmethacrylate) (poly-HEMA, Sigma), in the presence of treatments, were required. Cells were grown for 5 days and maintained in a humidified incubator at 37°C at an atmospheric pressure in 5% (v/v) carbon dioxide/air. After 5 days for culture, spheres > 50 μm were counted using an eye piece graticule, and the percentage of cells plated which formed spheres was calculated and is referred to as percent mammosphere formation. Mammosphere assays were performed in triplicate and repeated three times independently.

### Evaluation of mitochondrial mass and function

To measure mitochondrial mass by FACS analysis, cells were stained with MitoTracker Deep Red (Life Technologies), which localizes to mitochondria regardless of mitochondrial membrane potential. Cells were incubated with pre-warmed MitoTracker staining solution (diluted in PBS/CM to a final concentration of 10 nM) for 30-60 min at 37°C. All subsequent steps were performed in the dark. Cells were washed in PBS, harvested, re-suspended in 300 μL of PBS and then analyzed by flow cytometry (Fortessa, BD Bioscience). Data analysis was performed using FlowJo software (Tree star Inc.). Extracellular acidification rates (ECAR) and real-time oxygen consumption rates (OCR) for MCF7 cells were determined using the Seahorse Extracellular Flux (XFe-96) analyzer (Seahorse Bioscience). After exposure to Normoxia (21% O2) or Hypoxia (1% O2) for 96 h, 15, 000 MCF7 cells per well were seeded into XFe-96 well cell culture plates for 24h. Then, cells were washed in pre-warmed XF assay media (or for OCR measurement, XF assay media supplemented with 10mM glucose, 1mM Pyruvate, 2mM L-glutamine and adjusted at 7.4 pH). Cells were then maintained in 175 μL/well of XF assay media at 37C, in a non-CO2 incubator for 1 hour. During the incubation time, 5 μL of 80 mM glucose, 9 μM oligomycin, and 1 M 2-deoxyglucose (for ECAR measurement) or 10 μM oligomycin, 9 μM FCCP, 10 μM Rotenone, 10 μM antimycin A (for OCR measurement), were loaded in XF assay media into the injection ports in the XFe-96 sensor cartridge. Data set was analyzed by XFe-96 software after the measurements were normalized by protein content (SRB). All experiments were performed three times independently.

### ALDEFLUOR assay and separation of the ALDH positive population

ALDH activity was assessed by FACS analysis in MCF7 cells cultured for 72 h in Normoxia (21% O2) or Hypoxia (1% O2), as well as in MCF7 cells treated with Vehicle or Valproic Acid for 72 h. The ALDEFLUOR kit (StemCell Technologies) was used to isolate the population with high ALDH enzymatic activity by FACS (Fortessa, BD Bioscence). Briefly, 1 × 105 MCF7 cells were incubated in 1ml ALDEFLUOR assay buffer containing ALDH substrate (5 μl/ml) for 40 minutes at 37°C. In each experiment, a sample of cells was stained under identical conditions with 30 μM of diethylaminobenzaldehyde (DEAB), a specific ALDH inhibitor, as negative control. The ALDEFLUOR-positive population was established in according to the manufacturer’s instructions and was evaluated in 3 × 104 cells. Data analysis was performed using FlowJo software (Tree star Inc.).

### Label-free semi-quantitative proteomics analysis

Cell lysates were prepared for trypsin digestion by sequential reduction of disulphide bonds with TCEP and alkylation with MMTS. Then, the peptides were extracted and prepared for LC-MS/MS. All LC-MS/MS analyses were performed on an LTQ Orbitrap XL mass spectrometer (Thermo Scientific, San Jose, CA) coupled to an Ultimate 3000 RSLC nano system (Thermo Scientific, formerly Dionex, The Netherlands). Xcalibur raw data files acquired on the LTQ-Orbitrap XL were directly imported into Progenesis LCMS software (Waters Corp., Milford, MA, formerly Non-linear dynamics, Newcastle upon Tyne, UK) for peak detection and alignment. Data were analyzed using the Mascot search engine. Five technical replicates were analyzed for each sample type [10, 11].

### Evaluation of reactive oxygen species

Reactive oxygen species (ROS) production was measured by FACS analysis using CM-H2DCFDA (C6827, Life Technologies), a cell-permeable probe that is non-fluorescent until oxidation within the cell. MCF7 cells were cultured upon Normoxia (21% O2) or Hypoxia (1% O2) for 6 hrs or 96 hrs. Thereafter, cells were washed with PBS, and incubated at 37°C for 20 min with 1 μM CM-H2DCFDA, diluted in PBS/CM. All subsequent steps were performed in the dark. Cells were rinsed, harvested, re-suspended in PBS/CM and then analyzed by flow cytometry (Fortessa, BD Bioscience). ROS levels were estimated by using the mean fluorescent intensity of the viable cell population. The results were analyzed using FlowJo software (Tree star Inc.).

### Western blot analysis

MCF7 cell protein lysates were electrophoresed through a reducing SDS/10% (w/v) polyacrylamide gel, electroblotted onto a nitrocellulose membrane and probed with primary antibodies against HIF-1 R & D Systems phosphorylated ERK 1/2 (E-4), ERK2 (C-14), TOMM20 (F-10) and β-actin (C2), all purchased from Santa Cruz Biotechnology. Proteins were detected by horseradish peroxidase-linked secondary antibodies and revealed using the SuperSignal west pico chemiluminescent substrate (Fisher Scientific).

### Statistical analysis

Data is represented as the mean ± standard error of the mean (SEM), taken over ≥ 3 independent experiments, with ≥ 3 technical replicates per experiment, unless otherwise stated. Statistical significance was measured using the t-test. P ≤ 0.05 was considered significant.
